# The Application of Ni–Ti SMA Wires in the External Prestressing of Concrete Hollow Cylinders

**DOI:** 10.3390/ma14061354

**Published:** 2021-03-11

**Authors:** Aleksandra Dębska, Piotr Gwoździewicz, Andrzej Seruga, Xavier Balandraud, Jean-François Destrebecq

**Affiliations:** 1 Aldebud Aleksandra Dębska, 30-689 Kraków, Poland; aleksandra.debska@wp.pl; 2 Faculty of Civil Engineeering, Cracow University of Technology, 31-155 Krakow, Poland; aseruga@pk.edu.pl; 3 Institut Pascal, Université Clermont Auvergne, CNRS, SIGMA Clermont, 63000 Clermont-Ferrand, France; xavier.balandraud@sigma-clermont.fr (X.B.); j-francois.destrebecq@uca.fr (J.-F.D.)

**Keywords:** shape memory alloy, prestressing force, confinement, civil engineering, nickel–titanium

## Abstract

An innovative method for prestressing structural elements through the use of shape memory alloys (SMAs) is gaining increasing attention in research as this method does not require the use of mechanical anchorages for tendons. The activation of the memory effect by means of temperature variations (Joule effect) in effect produces high stresses in SMA components attached to concrete components as reported in the literature. This paper presents the work performed for the purpose of prestressing concrete hollow cylinders with the use of nickel–titanium (Ni–Ti) SMA wires. In the tests, a variety of hollow cylinders were made using the same concrete mix and with the same wall thickness (20 mm), but with different external diameters (200 mm, 250 mm, and 300 mm). Their prestressing was achieved by the means of Ni-Ti SMA wires of different diameters (1 mm, 2 mm, and 3 mm) wrapped around the cylinders. Longitudinal and circumferential strain during the thermal activation of the SMA wires by Joule heating was measured using gauges located on the internal surface of the hollow cylinders. The experimental protocol, recorded observations, and discussion of the effectiveness of the prestressing of concrete elements as a function of the test parameters are included in the text in detail. Comments on the conditions for effective prestressing of concrete cylinders with SMA wires are proposed in the conclusions of the paper.

## 1. Introduction

Technology relating to the prestressing of structures is widely known in civil engineering and is used mainly in the construction of bridges, tanks, industrial structures, and public buildings. At the current level of development of this method, prestressing techniques with conventional steel tendons (wires, strands, and bars) and tendons formed from composites developed in the last 30 years [1,2,3] are used. For the application of any of the proposed methods, it is necessary to use specific tensioning equipment usually in the form of hydraulic jacks, as well as appropriate types of anchorages for the given tendon type. For many years, steel tendons have been used for the building of new structures and for strengthening of existing constructions. In recent times, new methods of structural strengthening have been developed for historical monuments, using composite tapes and textiles, often made with carbon fibers as the principal component.

The use of shape memory alloys (SMAs) as another innovative technology used in the field of structural strengthening is an increasingly popular subject for considerations and developments. SMAs belong to the group of smart (intelligent) materials. Martensitic transformations triggered by temperature and stress occur in these metallic alloys. At zero stress, an SMA is in the austenite (A) phase at “high” temperatures, and it is in the martensite (M) phase at “low” temperatures. The A→M and M→A transformations also occur at constant “high” ambient temperatures upon mechanical loading and unloading, respectively. For various use of the SMA materials, as for example microactuators, a good understanding of the phase transformations and of the role of influencing parameters is important [4]. Whatever the type of activation (thermal or mechanical), a hysteresis of transformation is observed, making modeling and the use of SMAs complex tasks in engineering applications. The most famous property of SMAs promising for applications in civil engineering is the ability to regain the remembered geometry under thermal activation, referred to as the one-way shape memory effect [5,6,7]. Such geometry variations when properly steered may indeed be used for the generation of forces in structural elements.

Nickel–titanium (Ni-Ti) and, more generally, Ni–Ti-based alloys are the most frequently used SMAs in engineering applications, especially in the form of wires. In [8], for instance, it was reported that, during the return of an SMA material to its original shape from the deformed form, stress reaching even 800 MPa can be generated in the specimen in which strain is blocked. This conclusion is promising in the context of the future application of properly prepared material for the needs of force introduction in structural members; such pioneering applications were described in [9,10,11,12,13]. A more detailed approach to the martensitic transformation of Ni–Ti alloys includes also the presence of the R-phase during the cooling process, important for cyclic use of the material [14]. The introduction of prestressing with the use of this method usually as a single operation may be performed without the need for hydraulic pulling equipment at the place of prestressing, and it may, therefore, also be used in the locations where access is difficult.

The confinement of plain concrete cylinders using wound SMA wires is one of the proposed applications in civil engineering to create a state of compression; see, for example, [15,16,17,18,19,20,21]. The present paper describes the work performed for the purpose of prestressing hollow cylinders, which allows measurement of the strains on the inner surface while the SMA wire is wound on the outer surface. In the tests, hollow cylinders were made of the same concrete mix and with the same wall thickness (20 mm), but different external diameters (200 mm, 250 mm, and 300 mm). Their prestressing was achieved by the means of Ni–Ti SMA wires of different diameters (1 mm, 2 mm, and 3 mm) wrapped around the cylinders. Longitudinal and circumferential strains were measured using gauges located on the inner surface of the hollow cylinders during thermal activation of the SMA wires by Joule heating. Following the testing plan composed of a series of one-way, simple prestressing operations, the observations were focused on the effectiveness of the technique and did not include full analysis of the potential degradation of the SMA wires during the electrothermal working cycles as described in [22]. The experimental protocol, recorded observations, and discussion of the effectiveness of prestressing of concrete elements as a function of the test parameters are included in detail in the text. A description of the conditions for the effective prestressing of concrete pipes with SMA wires is proposed in the conclusions of the paper.

## 2. Properties of the SMA Wires Used

Ni–Ti SMA wires supplied by Nimesis Technology (Metz, France) were used in the present work to generate prestress states in concrete samples. Three wires with different diameters (1 mm, 2 mm, and 3 mm) were selected. Their chemical compositions were Ni_55.44_Ti (wt.%) for the 1 mm wire and Ni_55.84_Ti (wt.%) for the other two diameters of the wires. Table 1 presents the values of the four transformation temperatures for each wire diameter: austenite-start (A_s_), austenite-finish (A_f_), martensite-start (M_s_), and martensite-finish (M_f_). Values were obtained by means of dilatometric testing. Detailed information on the property testing of SMA wires can be found in [21,23,24]. They are the principal data needed for proper control of the phase transformation progress in SMA under varying temperature. It can be noted that M_s_ was negative and lower than A_s_ for the three wires. This property is not generic for SMAs. We selected this property for our SMA wires in order to have an ambient temperature T_amb_ between M_s_ and A_s_. The inequality M_s_ < T_amb_ < A_s_ is a key point of the study. Note that, by construction, the thermal hysteresis is high compared to most SMA wires. As a consequence, the SMA wires can also be “initialized” to the austenitic state by heating above A_f_, followed by a return to an ambient temperature higher than M_s_ [21,23]. Next, as a result of loading and further unloading of the austenitic SMA wire at the ambient temperature, a residual strain is generated in the wire. This residual strain is related to the transformation of the austenite into so-called oriented martensite upon loading (this is distinguished from the self-accommodating martensite, which is created by cooling and is not associated with a macroscopic strain). In these circumstances, the blocking of wire deformation and increasing its temperature above A_f_ lead to the generation of stress in the wire. This phenomenon is described in detail in, for example, [20]. For the mechanical characterization of each of the three SMA wires, a 20 kN Zwick 1455 laboratory testing machine was used to measure various mechanical parameters at 15 °C, starting from the austenitic state at zero stress: apparent Young modulus (E_SMA_) of the austenite, critical stress (σ_cr_) at which the A→M transformation starts, and the maximal stress (σ_max_) and residual strain (ε_res_) during the load-unload cycle to a maximum deformation of 6%. Lastly, the residual stress (σ_res_) at the blocked residual strain upon heating was measured. The mechanical properties of the three types of Ni–Ti wires used in the tests are shown in Table 1, in addition to the transformation temperature values. Details can be found in [24].

**Figure 1:**
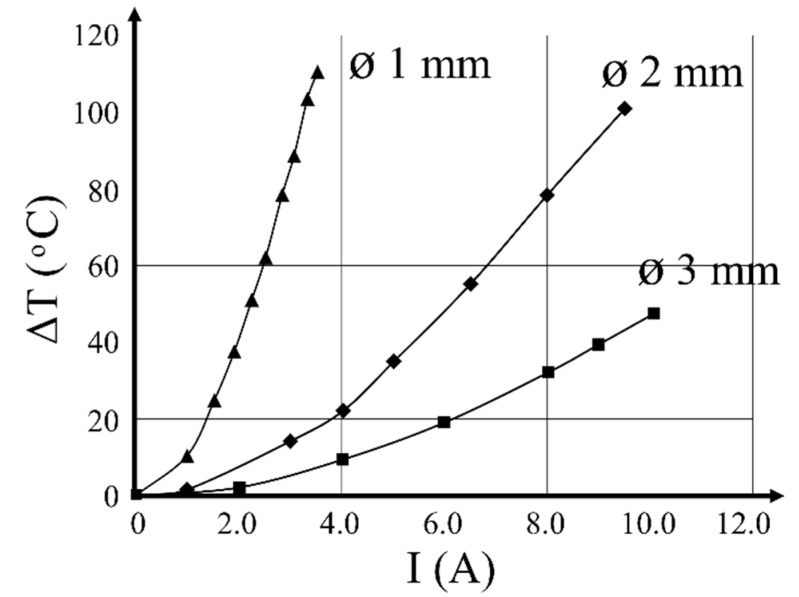
Temperature change ΔT vs. electric current I relationship in the three 200 mm long SMA Ni–Ti wires.

The Joule effect was used for heating the SMA wires. Heating tests were performed on 200 mm long wire specimens. Two parameters were recorded under different current intensities I (A): voltage U (V) and stabilized temperature variation ΔT (°C), using an infrared thermal camera (see [25] for details about the boundary conditions). Figure 1 shows the ΔT–I diagrams, which can be used for the purpose of defining the current value needed for the heating of the Ni–Ti wires to a given temperature. The equations for each diameter were as follows:

(1) $$\Delta T_{ø\;1\;\mathrm{mm}}=7.646{\;I}^{2}+5.047\;I$$

(2) $$\Delta T_{ø\;2\;\mathrm{mm}}=0.689{\;I}^{2}+2.400\;I$$

(3) $$\Delta T_{ø\;3\;\mathrm{mm}}=0.401{\;I}^{2}+0.729\;I$$

Prestressing of a structural member is generally a single operation. As a consequence, the tests were focused on the behavior of the specimens during the heating process of the SMA wire and the effectiveness of such a technique, while the cooling process was the finishing part for every test. Some influences, for example, the generation of the R-phase during cooling of the wire, which influences the wire resistance at this phase (as reported in [14]), were not considered. Following the same argument, considerations regarding the degradation of the material as a consequence of cyclic heating, as described, for instance, in [22], were also not included in the scope of the work.

## 3. Description and Preparation of the Concrete Samples

**Figure 2:**
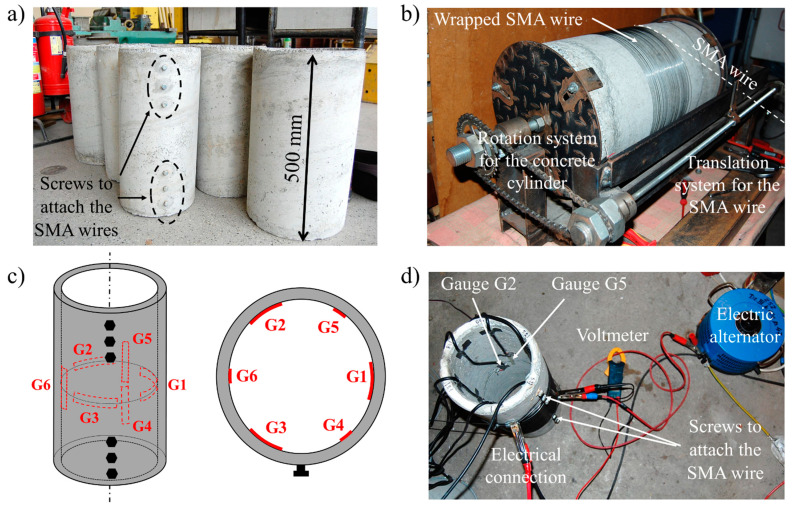
Test details: (**a**) photo of concrete test samples (hollow cylinders) after demolding, with anchoring bolts visible on one sample; (**b**) hand wire-winding machine; (**c**) location of the strain gauges on the internal surface of the concrete cylinders; (**d**) photo of the Joule effect setting for the prestressing of the hollow cylinder.

In the plan of this research, it was decided that the tests would be performed on three series of 500 mm long concrete hollow cylinders, each comprising three pieces with external diameters of 200 mm, 250 mm, and 300 mm (see Figure 2a). The measured values of wall thickness for the 200 mm cylinder were between 20.1 mm and 20.8 mm. They were between 22.1 mm and 22.9 mm for the 250 mm cylinder, and between 20.5 mm and 20.9 mm for the 300 mm cylinder. The variation coefficients υ of the mean wall thickness were between 3.7% and 9.6%.

All hollow cylinders were made of concrete composed of basalt gravel, the size of which was between 2 mm and 8 mm, and CEM I 32.5 R Portland cement (GÓRAŻDŻE CEMENT, Górażdże, Poland). The composition of the concrete mix per m^3^ was as follows: 353.4 kg of cement, 1202.6 kg of basalt gravel, 560 kg of river sand, and 213 w/c ratio of water, which was equal to 0.6. At the time of casting the test elements, several concrete specimens were collected for simultaneous verification of the mechanical properties of the material at the time of testing of the hollow cylinders. The dimensions of the material specimens were a diameter of 150 mm and a length of 300 mm for the plain cylinders, and the cubes were 150 × 150 × 150 mm^3^. After 7 days, the molds were disassembled and the concrete hollow cylinders (as well as the concrete specimens for material tests, see below) were protected with three layers of the colorless stretch film (LLDPE CAST) with thickness equal to 30 mic for a period of 28 days. For the sake of the estimation of the volumetric weight of concrete, all specimens were weighted and measured. The mean concrete weight obtained was 2465 kg/m^3^ with a coefficient of variation υ of 1.1%. The mean concrete compressive strength measured on the cubic specimens was f_c,cub_ = 51 MPa (υ = 8.1%). The mean value measured on the cylinders was f_c,cyl_ = 43 MPa (υ = 8.1%). The mean value of concrete elasticity modulus under compression measured at the level of 0.4 $f_{c,\mathrm{cyl}}$ was $E_{c}$ = 36,240 MPa (υ = 0.8%). On two cylindrical concrete specimens, the widened program of the test for the elasticity modulus was additionally performed with measurements taken at five levels of loading: 0.2, 0.3, 0.4, 0.5, and 0.6 $f_{c,\mathrm{cyl}}$. The test procedure followed the provisions given by Instytut Techniki Budowlanej (Building Research Insitute, Warszawa, Poland) [26]. The variations in concrete elasticity modulus following the force level are reported in Table 2. The mean value of concrete tensile strength under axial loading determined in the tests was 4.4 MPa (υ = 1.0%). The mean value of the concrete tensile strength determined in the splitting tests was 4.1 MPa (υ = 2.1%).

## 4. Procedure for the Creation of Prestress States in Concrete Hollow Cylinders with Use of SMA Wires 

The procedure presented below refers to the external prestressing of concrete cylinders with shape memory wires. The prestressing process was based on the thermal activation of the shape memory effect in the SMA wires. Heating of the material to a temperature exceeding the value of A_s_ was performed with the use of electric power. The prestressing procedure for every concrete cylinder was composed of the following four steps:

*Step I—Wire Preparation for Testing*

- The wire was subjected to heating until a temperature exceeding the value of A_f_ was obtained for the purpose of initialization in austenite phase.
- The wire was cooled to the ambient temperature. Let us recall that the ambient temperature was higher than Ms. As a consequence, transformation to martensite did not occur, and the material austenitic state was preserved. At this time, anchoring loops were formed on the wire ends.
- For part of the testing, controlled predeformation (residual strain) was applied to the SMA wire with the relevant pulling equipment.

*Step II—Winding and Anchoring of SMA Wires*

- The concrete specimen was installed in the specially designed winding apparatus shown in Figure 2b.
- The SMA wire loop was attached to the anchoring bolt on the external surface of the hollow cylinder, and the wire was wound on the surface.
- During the whole winding process, the ambient temperature was kept constant, and the SMA wire was protected from any temperature increase, especially from contact with hands.
- After winding, the wire end loop was fixed to the anchorage bolt.

*Step III—Connection of the Measurement Equipment*

- The following equipment was connected to the HBM QuantumX MX840A measuring amplifier with the use of shielded cables:
  ○ strain gauges installed on the internal surface of two hollow concrete cylinders—the tested cylinder and a reference cylinder (Figure 2c);
  ○ two temperature sensors, one for measuring temperature of the SMA wire $T_{\mathrm{ext}}$ and one for the internal surface of the concrete cylinder $T_{\mathrm{int}}$.
- An ammeter and voltmeter were used for monitoring the current parameters during the test.

*Step IV—Heating and Cooling of the Tested Element*

- At the initiation of the test, the SMA wire was connected to an electric current. An appropriate current source was used in dependence on the electric resistance of the SMA wire. The temperature increase in the SMA wire was controlled.
- After achieving the required temperature of the wire, the electrical current was disconnected and a temperature decrease was observed until it returned to the ambient temperature.

## 5. Standard Features of the Experimental Procedure

### 5.1. Setup of the Specimens and the Measurement Equipment

All tests in the research program were performed at the ambient temperature selected with consideration of the austenite start (A_s_) temperature for the given type of wire, which should not be exceeded. Part of the research program was, therefore, performed outdoors during winter. Low ambient temperatures and protection of the SMA wire from heating were an important advantage of this time of year.

The concrete deformation of every concrete cylinder was measured with strain gauges, which were installed at the mid-height of the inner surface of the hollow cylinders. Each of the specimens was equipped with six gauges: three in the longitudinal direction of the tube (vertically) and three in the circumferential direction, placed alternately along the internal circumference of the element. The gauges are denoted as follows and their positions are shown in Figure 2c:

- circumferential gauges—G1, G2, and G3;
- vertical gauges—G4, G5, and G6.

Temperature compensation was used due to the influence of temperature during prestressing of the tubes. Following the principles of the strain measurement theory, the gauges were connected in Wheatstone’s half-bridge system. During the tests, the following parameters were identified: ambient temperature ($T_{a}$), temperature of the pipe internal surface ($T_{\mathrm{int}}$), and temperature of the SMA wire wound on the pipe ($T_{\mathrm{ext}}$).

### 5.2. Application of Electrical Current for Prestressing of Concrete Hollow Cylinders

The testing stand was installed in a location where temperature varied between −1 °C and +10 °C. During all tests, the procedure described in Section 4 was used. The electrical current energy was transferred into thermal energy, which was reflected by the temperature increase in the wire through Joule’s law.

(4) $$P=I^{2}\;R=\frac{U^{2}}{R}$$

where P is the electric power (W) and R is the conductor resistance (Ω). Table 3 shows the values of the parameters required for fulfilling the condition of the temperature increase in the wire at 60 °C above the ambient temperature. Current density was calculated using Equations (1)–(3), and the resistance value was measured. Appropriate equipment for generating direct current was used. Resistance variations during the cooling period of the SMA wire (R-phase generation, as described, for instance, in [22]), as well as influence of the wire re-use on its resistance, were not considered.

### 5.3. Equations for the Analysis of the Combined Effect of Prestressing and Thermal Dilatation on Concrete Strain in Hollow Cylinders 

The influence of thermal deformation of concrete under conditions of the heating and cooling of the shape memory wire was considered. An analytical model was composed for the determination of forces acting in the SMA wire on the basis of measured deformation. It was assumed that the concrete circumferential strain measured with the strain gauges ε_c_ was linearly composed of the elastic strain provoked by prestressing with the SMA wire ε_c_,_SMA_ and the thermal strain ε_t_.

(5) $$\varepsilon_{c}=\varepsilon_{c,\mathrm{SMA}}+\varepsilon_{t}$$

According to the assumption that the material properties of concrete are constant in time and are independent of temperature, thermal strain in concrete is expressed as

(6) $$\varepsilon_{t}=\alpha_{t}\;\Delta T$$

where ΔT is the temperature variation, and α_t_ is the coefficient of thermal expansion for concrete (α_t_ = 10 × 10^−6^/°C).

Following the assumption of the mechanical isotropy of concrete, thermal isotropy was also assumed. Additionally, the temperature influence for any direction of deformation was separately considered in a linear manner. Therefore, the constitutive relationships between stress and strain were written in the following form, accounting for thermal deformation in accordance with a cylindrical coordinates system:

(7) $$\{\begin{array}{c} \varepsilon_{r}=E^{-1}\left[\sigma_{r}-\nu \left(\sigma_{c}+\sigma_{z}\right)\right]+\alpha_{t}\Delta T\\ \varepsilon_{c}=E^{-1}\left[\sigma_{c}-\nu \left(\sigma_{z}+\sigma_{r}\right)\right]+\alpha_{t}\Delta T\\ \varepsilon_{z}=E^{-1}\left[\sigma_{z}-\nu \left(\sigma_{r}+\sigma_{c}\right)\right]+\alpha_{t}\Delta T \end{array}$$

where ε_r_, σ_r_ are the strain and stress in the radial direction, ε_c_, σ_c_ are the strain and stress in the circumferential direction, and ε_z_, σ_z_ are the strain and stress in the longitudinal direction.

Using the Lame-Clapeyron approach for a thin cylindrical shell subjected to a radial uniform pressure p, it is possible to express the stress tensor σ.

(8) $$\left[\sigma \right]=\left[\begin{array}{ccc} \sigma_{r} & 0 & 0\\ 0 & \sigma_{c} & 0\\ 0 & 0 & \sigma_{z} \end{array}\right]$$

with

(9) $$\begin{array}{c} \sigma_{r}=-\frac{{p\;r}_{2}^{2}}{r_{2}^{2}-r_{1}^{2}}\left(1-\frac{r_{1}^{2}}{r^{2}}\right)\\ \sigma_{c}=-\frac{{p\;r}_{2}^{2}}{r_{2}^{2}-r_{1}^{2}}\left(1+\frac{r_{1}^{2}}{r^{2}}\right)\\ \sigma_{z}=0 \end{array}$$

where r is the radial distance from the centroid of the concrete hollow cylinder, and $r_{1}$ and $r_{2}$ are the internal and external radii of the concrete hollow cylinder, respectively.

It is possible to express the strain tensor ε as follows:

(10) $$\left[\varepsilon \right]=\left[\begin{array}{ccc} \varepsilon_{r} & 0 & 0\\ 0 & \varepsilon_{c} & 0\\ 0 & 0 & \varepsilon_{z} \end{array}\right]$$

with

(11) $$\{\begin{array}{c} \varepsilon_{r}=-\frac{1}{2\mu }\frac{{\mathrm{pr}}_{2}^{2}}{r_{2}^{2}-r_{1}^{2}}\left(\frac{\lambda +2\mu }{3\lambda +2\mu }-\frac{r_{1}^{2}}{r^{2}}\right)+\alpha_{t}\Delta T\\ \varepsilon_{c}=-\frac{1}{2\mu }\frac{{\mathrm{pr}}_{2}^{2}}{r_{2}^{2}-r_{1}^{2}}\left(\frac{\lambda +2\mu }{3\lambda +2\mu }+\frac{r_{1}^{2}}{r^{2}}\right)+\alpha_{t}\Delta T\\ \varepsilon_{z}=\frac{\lambda }{\mu \left(3\lambda +2\mu \right)}\frac{{\mathrm{pr}}_{2}^{2}}{r_{2}^{2}-r_{1}^{2}}+\alpha_{t}\Delta T \end{array}$$

where parameters μ and λ are classically expressed as functions of the Young’s modulus E and Poisson’s ratio ν of concrete as follows:

(12) $$\{\begin{array}{c} \mu =\frac{E}{2\left(1-\nu \right)}\\ \lambda =\frac{\nu E}{\left(1+\nu \right)\left(1-2\nu \right)} \end{array}$$

As a function of the local equilibrium of the cylindrical shell, the pressure p applied by the SMA wires on the external surface of the hollow cylinder is expressed in Equation (7).

(13) $$p=\frac{{\pi \;d}^{2}\;\sigma_{\mathrm{SMA}}}{2\;e\;\left(2r_{2}+d\right)}\geq 0$$

where $\sigma_{\mathrm{SMA}}$ is the longitudinal stress in the SMA wire, d is the diameter of the SMA wire, and e is the axial spacing of the SMA wire (pitch) wound on the concrete hollow cylinder.

## 6. Results of the Performed Tests 

### 6.1. Results Obtained from Tests of Concrete Hollow Cylinders Prestressed with SMA Wire Without Preliminary Deformation

Nineteen tests were successfully performed on concrete hollow cylinders prestressed with wound SMA wires that were not pretensioned and not preliminarily deformed (not predeformed). Information on the number of tests performed for the various SMA wire diameters used and the various diameters of concrete specimens is presented in Table 4. For practical reasons, lengths of Ni–Ti wires measuring 28.80 m were prepared for this purpose.

**Figure 3:**
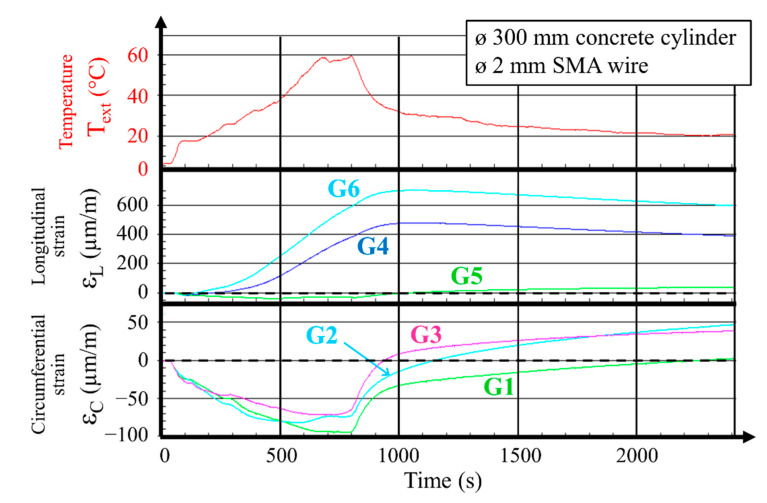
Measurements of wire temperature Text, longitudinal strain εL, and circumferential strain εc of concrete during the process of testing a 300 mm diameter hollow cylinder wound with a 2 mm diameter SMA wire without predeformation (circumferential gauges—G1, G2, and G3; longitudinal gauges—G4, G5, and G6). Note that gauge G5 was not working for all tests without predeformation.

Figure 3 presents example results of the measurements which were recorded during the heating and cooling process of the specimen composed of a concrete cylinder with a diameter of 300 mm wound with Ni–Ti wire with a diameter of 2 mm. Three diagrams were prepared as a time function: a diagram of the wound wire temperature, a diagram of the vertical concrete deformation, and a diagram of the circumferential concrete deformation. The temperature of the internal surface of the cylinder $T_{\mathrm{int}}$ and ambient temperature $T_{a}$ were also measured.

For the first testing phase (heating), the temperature increased to +60 °C. With a wire temperature increase of 20 °C (200 s after current connection), the initiation of an increase in strain was observed in the longitudinal direction of the hollow cylinder. It is worth noting that the result of the thermal dilatation of concrete was included in all measurements of the vertical (longitudinal) and circumferential strain gauges. The duration of the electric current flow through the wound wires was 800 s. It may be observed in the concrete deformation diagrams that, during the initial 200 s of the heating period, the vertical (longitudinal) concrete strain was not significant, while the circumferential compressive concrete strain increase was substantial and reached a level of −50 µm/m. The increase in concrete strain in the circumferential direction provided evidence of the martensite transformation in the SMA wire and was consequently also evidence of concrete prestressing. After 800 s of heating (when the maximal wire temperature of 60 °C was reached), the mean circumferential compressive strain in the concrete was at a level of around −85 µm/m, and the mean strain in the longitudinal direction was around 333 µm/m (at this moment, the temperature of the concrete was lower than the wire temperature).

After disconnecting the electrical current, the SMA wire quickly lost its temperature while concrete retained its heat for much longer. During the cooling period, compressive strain in concrete was progressively lost. The maximal observed main longitudinal strain increase in the concrete was around 400 µm/m after 1000 s (SMA temperature T_ext_ = 32 °C). It is understood that the concrete temperature was higher than wire temperature. After 1300 s, the mean circumferential strain in concrete approached zero and, with further time, the strain became positive. The explanation for such a development is that the residual strain in the wire decreased to zero. After 2400 s, the wire temperature was 20 °C, the mean longitudinal strain in concrete was 333 µm/m, and the mean circumferential concrete strain was 30 µm/m.

According to the results, it can be concluded that the prestressing effect obtained with the SMA wire without preliminary deformation was not stable over time. A comment can be made about the heating phase. Before winding the SMA wire, it was in the austenite state; however, a low quantity of the oriented martensite probably appeared due to the curvature of the wire when installed on the external surface of the concrete hollow cylinder. During the temperature increase above the A_f_ value, the shape memory effect was activated, thus generating a (relatively low) circumferential compressive strain increase in concrete. A similar effect was previously reported in [16] for plain concrete cylinders. The loss of effect upon cooling can be explained by the appearance of oriented and self-accommodating martensite. In any case, in spite of the number of tests, the observed effect was found to be not sufficient for the permanent prestressing of the concrete hollow cylinders. Accordingly, it was decided to perform further tests exclusively with the preliminary deformation of SMA wires and to resign from the presentation of remaining results captured during tests with use of wires that were not predeformed.

### 6.2. Concrete Hollow Cylinders Prestressed with the Preliminary Deformed SMA Wire 

#### 6.2.1. Experimental Setup

In the next step of the testing program, the effectiveness of the prestressing of concrete hollow cylinders with predeformed Ni–Ti coil was verified. Ni–Ti wires measuring 28.80 m in length were prepared for this purpose.

The experiments were performed with the use of all nine concrete hollow cylinders (three hollow cylinders for each of the wire diameters). Eighteen tests were performed. During the preparation of every test, the SMA wire was predeformed and was then wound over the external surface of the hollow cylinder. After every test, the SMA wire was unwound and was then heated above A_f_ in order to retrieve the austenite state; it was then predeformed and wound again on a hollow cylinder. As a consequence of the differences between the diameters of the hollow cylinders, the number of possible windings with the wire of a constant length varied as follows for each of the hollow cylinders:

- For the hollow cylinder with an external diameter of 200 mm, there were 45 windings of the wire placed in the central strip of the hollow cylinder surface, which amounted to a width of approximately 0.17 m.
- For the hollow cylinder with an external diameter of 250 mm, there were 38 windings of the wire placed in the central strip of the hollow cylinder surface, which amounted to a width of approximately 0.15 m.
- For the hollow cylinder with an external diameter of 300 mm, there were 32 windings of the wire placed in the central strip of the hollow cylinder surface, which amounted to a width of approximately 0.13 m.

The pitch of the spiral coil was independent of the winding precision. The nominal pitch was 4 mm. As with the test without predeformation, the following parameters were recorded during the tests: ambient temperature (T_a_), temperature of the internal surface of the hollow cylinder (T_int_), and temperature of the wire wound on the hollow cylinder (T_ext_).

All tests of this series were performed following the same procedure. In contrast to the tests described in Section 6.1, all the SMA wires were predeformed before winding, as explained in Section 4. The initial elongation of the wires was set at two levels: 3% and 6%. The measurement equipment was connected to the tested cylinder and to the reference cylinder, and the hollow cylinder prestressing was performed using a flow of electrical current through the SMA material winds, as presented in Figure 2d.

#### 6.2.2. Test Results and Stresses Calculated in the SMA

**Figure 4:**
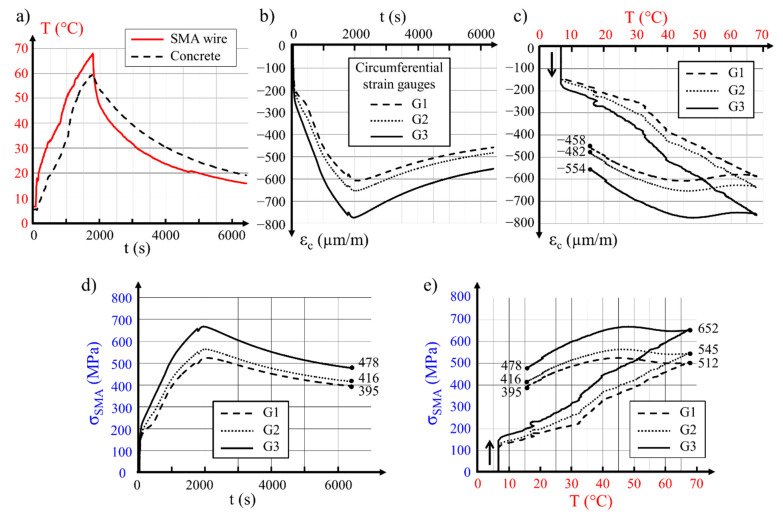
Results recorded during testing of the 300 mm hollow cylinder prestressed with predeformed 2 mm Ni–Ti wire: (**a**) temperature of the wire and the concrete surface over time; (**b**) variations of circumferential concrete strains over time; (**c**) variations of circumferential strain over concrete in relation to wire temperature; (**d**) stress in Ni–Ti wire in relation to time; (**e**) stress in Ni–Ti wire as a function of temperature.

As explained in Section 5.1, variations of concrete strain in the circumferential direction (three strain gauges) and in the longitudinal direction (three strain gauges), as well as the temperature of the wire and that of the internal hollow cylinder surface, were monitored and recorded during each test. The measured values are presented as diagrams of strain and temperature, both in relation to time. Figure 4 presents example diagrams determined during the prestressing tests performed on the 300 mm diameter concrete hollow cylinder with a 2 mm SMA wire. The main features shown on the diagrams are discussed below. Detailed results for the same specimen are included in Table 5 No. 16. Variations of the temperature during the whole test are presented in Figure 4a. The temperature increase activated the martensite-to-austenite transformation. Let us recap that the start and finish transformation temperatures at zero stress were A_s_ = 2 °C and A_f_ = 13 °C, respectively (see Table 1). As a consequence, transformation started nearly instantaneously after the initiation of the flow of electrical current. This phenomenon can be seen in Figure 4b,c. The circumferential strain in concrete resulting from the clamping of the Ni–Ti wire around the hollow cylinder started with a sharp increase at a wire temperature of 7 °C (Figure 4c). Following further temperature increases, growth of the compressive strain in concrete was registered. After reaching 67 °C in the tested wire, the current was disconnected. The moment of current disconnecting may be found in the diagram showing the relationship between the circumferential strain in concrete and time (Figure 4b) in the form of a small discontinuity in any line of captured values. A decrease in the wire temperature to a level of 40 °C did not substantially influence the circumferential strain generated in concrete (Figure 4c). Further cooling of the specimen to ambient temperature provoked a partial loss of the generated concrete deformation.

According to the concrete strain evolution shown in Figure 4b,c, and on account of Equations (8)–(13) presented above, diagrams of stress development in the 2 mm Ni–Ti wire were built as functions of time and temperature. In Figure 4d, an instantaneous stress increase to the level of 200 MPa may be observed, which resulted from a quick activation of the shape memory effect. Further increases in the wire temperature allowed the progress of the transformation of oriented martensite into austenite. This progress provoked a further increase in stress in the SMA wire. A partial loss of the wire stress initially generated during heating time was observed after a decrease in its temperature to below 40 °C (Figure 4e).

Analysis of the recorded measurements led to the conclusion that, during cooling time, nearly 24% of the maximal stress obtained in the test for the material was lost. Such a loss was a consequence of the thermomechanical properties of the shape memory alloy used and could be qualified as a non-standardized initial loss of the prestressing force. The decrease in residual stress in the prestressing wire was estimated at 6.5%, while accounting for the influence of temperature variations on concrete deformation.

## 7. Analysis of Test Results 

### 7.1. Discussion of the Results from Tests and Analysis 

The registered results of the tests were used for the determination of prestressing effectiveness. Measurements were taken at the final stage of the tests of the 18 concrete hollow cylinders, including concrete strain in the circumferential and longitudinal directions, and temperatures of the surrounding air T_a_, of the wire T_ext_, and of the internal surface of the hollow cylinder T_int_ are included in Table 5 Additionally, the stress values were analytically determined.

The following parameters were used for the calculations of stress and force in the SMA wires: E_c_ (elasticity modulus for concrete, equal to 36 GPa), T_ext_ (SMA wire temperature measured in the final step of the test), T_int_ (temperature of the internal surface of the concrete hollow cylinder measured in the final step of the test), T_a_ (ambient temperature of the test), and ε_c_ (circumferential strain in the concrete measured with the strain gauges in the final step of the test). With the use of Equations (8)–(13), after excluding the thermal dilatation of the concrete, the following quantities were calculated for every concrete hollow cylinder prestressed with the SMA wires:

P—pressure [MPa];
σ_c,SMA_—circumferential compression stress in concrete at the residual state [MPa];
σ_res_—stress in shape memory wire at the residual state [MPa];
F_SMA_—force in SMA wire remaining after prestress execution [N].

The mean values of the circumferential stress in concrete caused by the prestressing wire action (σ_c, SMA_) and mean values of the residual stress in the prestressing wire σ_res_ calculated on the basis of measurements are included in Table 5.

The effective force in the SMA wire depended on its diameter. It can be observed also that, for a part of the cylinders prestressed with the wire with a diameter of 3 mm (No. 13 and 17), the force in SMA wire was much lower than for the remaining specimens of the same type (No. 12 and 18, respectively). Such lower effectiveness was mainly related to the difficulty in blocking the wire ends at the beginning of the test. An additional influence may be related to degradation of the SMA wire by the flow of electrical current. The value of the electric current (equal to 13 A) used in these tests was much greater than the value influencing the properties of SMA wires, as reported, for example, in [22]. A summary of the detailed results is presented in Table 6 and Table 7. For all types of specimens, defined by the hollow cylinder diameter and wire diameter, the mean values of the principal results are included. The mean values of the circumferential concrete strain and stress, as well as the estimated force in the SMA wire at the residual state (after activation of the shape memory effect and cooling to the ambient temperature), are summarized.

### 7.2. The Influence of Preliminary Wire Deformation on Prestressing Effectiveness 

For the investigation of the relationship between the value of the initial predeformation of the wire and the level of prestressing obtained in the concrete hollow cylinders, four specimens with external diameters of 250 mm prestressed with Ni–Ti wire with a diameter of 2 mm were tested. The initial predeformation was at two different values (6% and 3%); there were two cylinders for each predeformation value. Tests I and II refer to the initial predeformation of 6%, while tests III and IV refer to the 3% predeformation.

**Figure 5:**
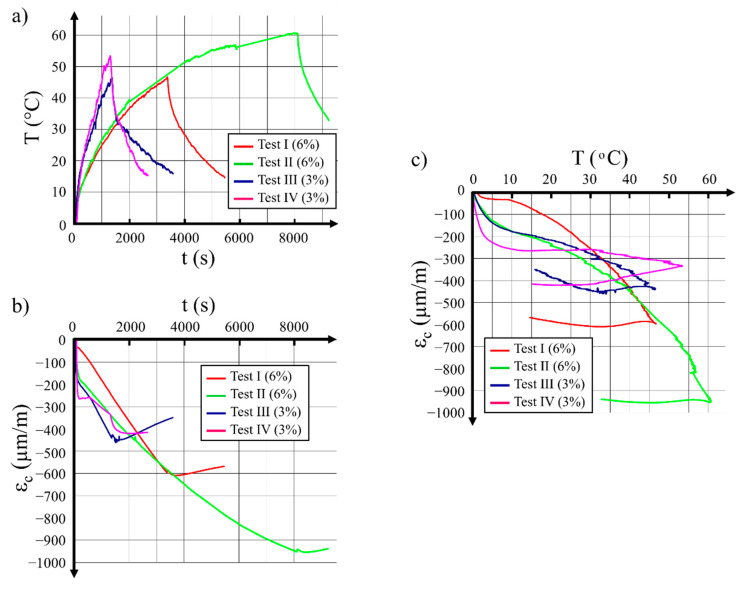
Results captured during tests with Ni–Ti wire with a diameter of 2 mm wound on the 250 mm diameter pipe: (**a**) wire temperature variations over time during electric heating, then cooling; (**b**) variations of circumferential concrete strain over time; (**c**) variations of circumferential concrete strain as a function of temperature. Note that measurements for test II were stopped before the specimen was cooled to the ambient temperature.

After the initial predeformation, the wire was wound on the hollow cylinder and was connected to the electrical current. A diagram of wire temperature variations over time during the heating and cooling periods is presented in Figure 5a for the four tests. It may be observed that, after connecting the electrical power, the wire predeformed to 3% heated much faster than the wire predeformed to 6%. The time needed for heating the wires to 45 °C for tests I and II was 3000 s, which was three times more than the time needed for the heating of specimens III and IV.

Variations of the circumferential concrete strain as a function of SMA wire heating time for the four tests are shown in Figure 5b. It is observed that the strain increase in the first 1500 s of heating was similar for all four tests. Afterward, the concrete strain in tests III and IV decreased as an effect of the wire cooling, while, for tests I and II, the concrete strain increased during the whole period of heating of the longer wire.

Variations of the concrete strain in the circumferential direction related to temperature for the four tests described above are presented in Figure 5c. For the sake of comparison, all concrete strain values were captured for the SMA wire temperature of 45 °C.

Test I (initial predeformation 6%), −545 μm/m;
Test II (initial predeformation 6%), −547 μm/m;
Test III (initial predeformation 3%), −428 μm/m;
Test IV (initial predeformation 3%), −305 μm/m.

The results collected during the tests performed allowed the following conclusions to be formulated:

- SMA wire predeformed to a higher initial strain value needed a longer duration of electrical current flow to reach the expected temperature. In the case of wire with a diameter of 2 mm predeformed to 6%, the time needed to reach a temperature of 45 °C was three times longer than the time needed to heat the same wire predeformed to 3%.
- As a consequence of the longer heating time required for the wire predeformed to 6%, the concrete hollow cylinder was also heated more and this provoked an additional thermal deformation of the concrete.
- At a wire temperature of 45 °C, the circumferential strain of the concrete in the hollow cylinder prestressed with the wire predeformed to 6% was around 50% higher than the strain in the concrete hollow cylinder with the wire predeformed to 3%; the measured concrete strain included the influence of prestressing with the SMA wire and the thermal deformation of the concrete.
- It can be seen in Figure 5c that, for the hollow cylinder prestressed with wire subjected to 6% predeformation, a higher final strain was preserved after cooling, and this meant that the residual stress (σ_res_) in this wire was higher than in the case of a wire predeformed to 3%.

## 8. Comments on Tests Conditions 

### 8.1. Conditions for Performing the Prestressing of Concrete Hollow Cylinders with the Use of SMA Wires Activated with an Electrical Current

During the tests, attention was paid to the proper registration of the concrete strain and the temperature of the internal surface of the hollow cylinders, as well as that of the wire. The results were used for the determination of the final stress in the concrete hollow cylinders in the circumferential direction and the residual stress σ_res_ in the wound wire. A constant pitch of the wound wires of 4 mm was successfully achieved in order to exclude any risk of electrical short cirquit. Nevertheless, the following potential imperfections of the tests were observed:

1. Limited space between the wires eliminated the possibility to install temperature sensors on the external surface of the hollow cylinders. For the needs of analysis, it was assumed that the external surface temperature was equal to the temperature of the wound wire—such an assumption is rather rough, as contact between the wires and the concrete is not uniform.
2. As a consequence of the above point, the external concrete surface was not heated evenly.
3. During the execution of the prestressing of the hollow cylinders, their position was vertical and this could have inhibited the ventilation and cooling of the internal surface.

**Figure 6:**
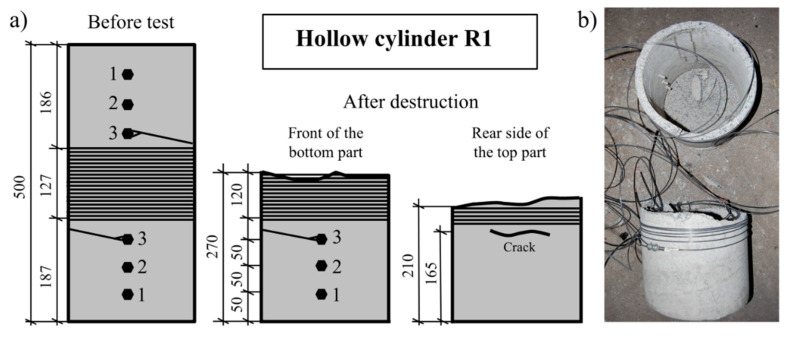
R1 hollow cylinder before and after its destruction: (**a**) drawing of the specimen before destruction, with front of the bottom part and rear side of the bottom part after destruction; (**b**) photo of the R1 specimen after its destruction. Units in (mm).

The speed of increase in the wire temperature under the influence of current flow depended on the type of wire, as well as its diameter. Together with the temperature of the wires, the temperature of the concrete cylinders also increased. The progress of these variations depended on the current parameters, the wire impedance, the wire and cylinder thermal capacity, and the ambient temperature. Diagrams of the temperature increase of the wire and concrete above the initial level registered during prestressing of the hollow cylinders are presented in Figure 6. In each case, after reaching the required temperature level, the electrical current was disconnected and the temperature of the wire and concrete decreased. ΔT is the increase in temperature of the internal surface of the hollow cylinder and of the wire from their initial levels. Every temperature measurement was started before connection of the SMA wire to the current source.

The temperature variation of the Ni–Ti wire with a diameter of 1 mm was the most rapid of all wire diameters. The maximal value of the wire temperature was reached after 6 to 10 mins of heating. At the same time, the temperature of the internal surface of the concrete remained low (10 °C to 30 °C); thus, the temperature gradient between the SMA wire and the internal concrete surface could reach around 75 °C after 3 mins of heating (as was the case for the hollow cylinder with a diameter of 250 mm). After 5 mins, this gradient dropped to 30 °C. For all specimens, the temperature of the wire decreased quickly after the end of heating, while the temperature of concrete after reaching its maximum decreased less quickly. For the hollow cylinders with a diameter of 200 mm, the temperature of the concrete 7 mins after the test was higher than the temperature of the wire. This may be explained by the lowest thermal capacity of the 200 mm concrete cylinder from all specimens. For hollow cylinders of 250 mm and 300 mm prestressed with the 1 mm diameter wire, the temperature of the wire and of the concrete became equal after 20 mins, while, for the 200 mm cylinder, the concrete temperature was higher for a much longer period of time.

Prestressing of the concrete hollow cylinders with SMA wires with diameters of 2 mm and 3 mm required a substantially longer heating time, as, under the effect of lower electrical impedance of the material, less heat was released. A heating time of 20 to 30 mins also resulted in a systematic increase in temperature of the internal surface of the hollow cylinder. The temperature gradient during the heating time did not exceed 25 °C (Figure 6b,c). At the moment when the current was disconnected, a sudden drop in wire temperature in the range of 20 °C was observed, followed by further cooling. Measurements of the concrete surface temperature did not reveal any instantaneous drop in temperature. This shows that the progress of the cooling of the concrete was slow and the temperature measured on the internal surface of the hollow cylinder was at most 20 °C higher than the wire temperature for the whole time until the end of the test. A similar observation was noted for the cylinders of 250 mm and 300 mm as for the 200 mm cylinder prestressed with 1 mm wire; the concrete temperature decreased slowly and wire temperature was lower during cooling time. This is understood as the influence of the thermal capacity of the parts of the specimen. 

### 8.2. The Failure of Two Hollow Cylinders during the Tests

During the execution of the entire testing program, two failures of concrete hollow cylinders occurred, as is typical for a new direction of research; two hollow cylinders (R1 and R2), each with an external diameter of 300 mm, broke during prestressing. In the first case, hollow cylinder R1 (Figure 6) was subjected to prestressing with Ni–Ti wire with a diameter of 3 mm which was predeformed to the initial deformation level of 6%. After winding the wire over the hollow cylinder, the prestressing operation commenced. Direct electrical current with density I = 15 A and voltage U = 50.8 V was used in this case. The whole test took only 350 s (including 280 s of heating time). During this time, the wire temperature increased by ΔT = 9.0 °C (quantity of the released heat Q = 213 kJ).

**Figure 7:**
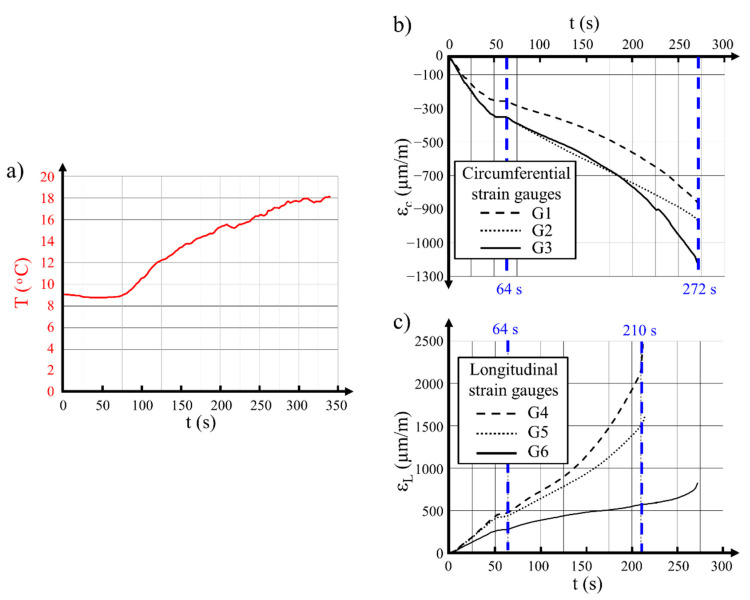
Results recorded during testing of the 300 mm hollow cylinder prestressed with 3 mm SMA wire—specimen R1: (**a**) temperature over time for the Ni–Ti wire; (**b**) circumferential strain for concrete; (**c**) longitudinal strain for concrete.

The wire temperature increase in relation to time and the variations of concrete strain on the internal surface of the hollow cylinder in the longitudinal and circumferential directions are shown in Figure 7. Analysis of the concrete strain variations presented in Figure 7b,c allowed us to state that, at 64 s from the start of the test, when the electric current was connected, the concrete strain started to grow linearly until 125 s, and a further more intensive strain increase in the longitudinal direction captured by strain gauges G5 and G4 was observed. The two strain gauges G5 and G4 were damaged nearly simultaneously after 210 s of the test, reaching strain at the levels of 1500 and 2100 µm/m, respectively (Figure 7c). This meant that, at the location of the strain gauges, concrete cracking appeared in the circumferential direction, perpendicular to the longitudinal direction of the strain gauges (Figure 6).

During further execution of the test program, concrete strains recorded with the strain gauges installed in the circumferential direction linearly increased to 250 s. After this moment, a significant increase in the strains registered by gauges G1 and G3 was observed. Destruction of all strain gauges in the circumferential direction and in one longitudinal direction (G6) occurred simultaneously at 275 s. The upper part of the hollow cylinder separated from the lower part with a bang and was thrown to a height of around 1.5 m. Loosened coils of the Ni–Ti wire absorbed its kinetic energy, and the separated part fell to the floor. The Ni–Ti wire was neither broken nor damaged (Figure 6b).

**Figure 8:**
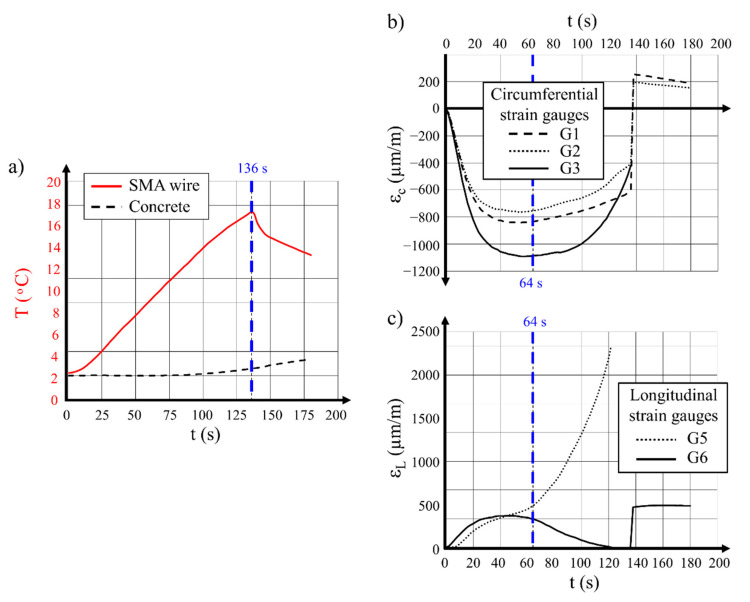
Results recorded during testing of the 300 mm hollow cylinder prestressed with 3 mm SMA wire—specimen R2: (**a**) temperature variations of the wire and of the concrete specimen internal surface over time; (**b**) strain variations of concrete in the circumferential direction over time; (**c**) strain variations of concrete in the longitudinal direction over time.

In the other case, the hollow cylinder R2 was destroyed during prestressing with the same diameter of Ni–Ti wire (3 mm). The specimen was prepared according to the procedure described in Section 4. Wire predeformed to 3% was wound on the hollow cylinder. An electrical current with a density of I = 20 A and a voltage of U = 70 V was connected. The temperature variations over time measured for the wire and for the internal surface of the hollow cylinder (Table 5, No. 18) are presented in Figure 8a.

Analysis of the presented diagrams led to the conclusion that the wire was quickly heated to a temperature of 38.7 °C, while, on the internal surface of the concrete hollow cylinder, the temperature was nearly stable for a period of 100 s and remained at a level of + 5 °C. After 136 s from the start of the test, the difference between temperatures on the external and internal surfaces of the hollow cylinder was 32 °C. At this time, the hollow cylinder burst was observed 0.21 m from the bottom. The final temperatures of the wire and concrete were 26 °C and 5 °C, respectively.

A careful analysis of the strain development in concrete in both measured directions shown in Figure 8b,c brought clear information that an important moment in the test occurred after 64 s of wire heating. At this time, the circumferential strain reached its maximal value and the longitudinal strain started to rapidly increase or decrease.

The above-described conditions were characteristic of the moment of creation of the circumferential cracks, perpendicular to the longitudinal direction of the hollow cylinder. The temperature difference (gradient) between the external and internal surfaces of the concrete at 64 s was 16 °C. Considering the value of concrete elasticity modulus at the level of 36 GPa, this gave a tensile stress in concrete of 5.9 MPa. The estimated value was higher than the concrete tensile resistance in the axial load, and it was 4.4 MPa on the basis of laboratory tests.

To conclude, it is expressed that hollow cylinder failures were provoked by stress exceeding concrete tensile resistance. Thermal actions generated at the time of cylinders prestressing, together with the load applied to hollow cylinders by means of wound wire with prestressing force activated within it, were the cause of the damage.

## 9. Conclusions

As a result of the performed research, the following conclusions can be drawn:

- Cylindrical concrete elements may be effectively prestressed with use of initially predeformed wire formed from shape memory alloy (SMA), in which prestressing is activated in the method of heating the wires with a flow of electrical current.
- Values of the circumferential compressive stress in concrete σ_c_, as well as values of permanent stress in the SMA wires σ_res_, depend on the type and diameter of the wire, the initial predeformation value, and the hollow cylinder diameter and thickness.
- Prestressing of the concrete hollow cylinders with SMA wires with a higher level of initial deformation (6%) is more effective as it brings higher values of circumferential compression stress in concrete σ_c_, as well as higher values of permanent stress in the prestressing wire σ_res_, than for the specimens with wire predeformation of 3%.
- The performed testing program showed that activation of the shape memory effect in the wires with use of a flow of electrical current is effective in practical usage. The selection of the Ni–Ti wire diameter should relate to the conditions of the prestressing execution. It should be noted that wire predeformed to 6% requires a much longer action of electrical current than wires predeformed to 3%. Longer heating of SMA wires also results in the heating of concrete, and this may lead to thermal deformation of the concrete.
- Too much heat in the wire may result in a high thermal gradient in the hollow cylinder wall, which may consequently provoke high tensile stress in concrete; such conditions may provoke exceeding of the concrete tensile strength and, ultimately, the failure of the hollow cylinder. Overheating may also damage the SMA wire.

## Figures and Tables

**Table 1:** Properties of the three shape memory alloy (SMA) wires: transformation temperatures and mechanical properties from [21,23].

**SMA Wire Diameter**	**Transformation Temperatures at Zero Stress**				**Mechanical Characteristics for a Load–Unload Test at 6% Maximum Strain at 15 °C, Followed by Heating at Fixed Residual Strain** **ε_res_**				
	**M_f_**	**M_s_**	**A_s_**	**A_f_**	**E_SMA_**	**σ_cr_**	**σ_max_**	**ε_res_**	**σ_res_**
	**(°C)**	**(°C)**	**(°C)**	**(°C)**	**(GPa)**	**(MPa)**	**(MPa)**	**(%)**	**(MPa)**
ø 1 mm	−51	−7	14	22	34.7	244	269	5.1	202
ø 2 mm	−66	−11	2	13	21.7	314	309	4.6	248
ø 3 mm	−51	−30	1	10	15.5	340	349	3.0	179

**Table 2:** Modulus of elasticity of concrete versus the level of loading.

**Sample No.**	$\mathrm{Modulus}\;\mathrm{of}\;\mathrm{Elasticity}\;E_{c}$ **(MPa)**				
	**$0.2\;f_{c,cyl}$**	**$0.3\;f_{c,cyl}$**	**$0.4\;f_{c,cyl}$**	**$0.5\;f_{c,cyl}$**	**$0.6\;f_{c,cyl}$**
1	38,240	37,860	36,170	35,300	34,100
2	39,130	37,100	36,000	34,820	33,870

**Table 3:** Parameters required for performing the SMA wire heating tests with direct current.

**Wire Diameter**	**L (m)**	**ΔT (°C)**	**R (Ω)**	**I (A)**	**P (W)**	**U (V)**
ø 1 mm	40	60	41.6	2.5	260	104
ø 2 mm	80	60	20.8	7	1041	147
ø 3 mm	80	60	9.3	13	1562	120

**Table 4:** Number of tests performed on concrete hollow cylinders with use of SMA wires without preliminary deformation.

**Wire Diameter**	**External Pipe Diameter**		
	**200 mm**	**250 mm**	**300 mm**
ø 1 mm	4 tests	1 test	2 tests
ø 2 mm	4 tests	1 test	2 tests
ø 3 mm	2 tests	1 test	2 tests

**Table 5:** Results of measurements and calculation of mean forces in SMA wires wound on the concrete cylinders.

**No.**	**ΔL/L** **(%)**	**T_a_** **(°C)**	**T_ext_** **(°C)**	**T_int_** **(°C)**	**ε_c1_** **(10^−6^)**	**ε_c2_** **(10^−6^)**	**ε_c3_** **(10^−6^)**	**ε_c,av_** **(10^−6^)**	**ε_t_ =α ΔT** **(10^−6^)**	**ε_c,SMA_** **(10^−6^)**	**p** **(MPa)**	**σ_c.SMA_** **(MPa)**	**σ _res_** **(MPa)**	**F_SMA_** **(N)**
**Pipe ø 200 mm–SMA wire ø 1 mm**														
1	3	−3	13.3	22.5	−155.9	−143.2	−151.9	−150.3	−91.8	−58.5	0.38	−2.1	194.2	152.5
**Pipe ø 200 mm–SMA wire ø 2 mm**														
2	3	4	20.1	27.4	−420.6	−450.2	−488.0	−452.9	−72.6	−308,3	2.46	−13.8	317.1	995.7
3	3	−1	20.3	26.0	−615.7	−486.9	−537.2	−546.6	−57.0	−483.6	3.17	−17.6	408.2	1281.8
4	3	3	52.9	41.1	−388.8	−754.6	−420.3	−521.2	117.8	−639.0	3.38	−18.8	435.5	1367.4
5	3	2	20.4	26.2	−537.7	−486.9	−615.8	−546.8	−57.8	−489.0	3.17	−17.6	407.7	1280.2
6	3	7	21.2	30.1	−298.9	−383.8	−319.8	−334.2	−89.0	−245.2	1.59	−8.8	204.4	641.8
**Pipe ø 200 mm–SMA wire ø 3 mm**														
7	3	3	14.5	19.9	−727.1	−662.5	−467.3	−618.9	−54.5	−564.4	3.66	−20.3	210.2	1485.0
8	3	4	24.6	38.8	−724.9	−458.1	−683.6	−607.2	−141.0	−466.2	3.02	−16.8	173.5	1225.7
**Pipe ø 250 mm–SMA wire ø 1 mm**														
9	4	−1	19.6	24.9	−137.5	−135.3	−129.9	−134.3	−52.7	−81.6	0.43	−2.9	276.4	217.0
**Pipe ø 250 mm–SMA wire ø 2 mm**														
10	3	4	24.1	37.0	−344.5	-	−415.9	−380.2	−129.0	−251.2	1.33	−9.0	213.7	670.9
11	4	0	20.2	25.0	−295.7	−412.5	−341.4	−349.9	−47.8	−302.1	1.6	−10.9	256.9	806.7
**Pipe ø 250 mm–SMA wire ø 3 mm**														
12	4	−1	19.6	24.9	−514.3	−683.3	−549.0	−582.2	−52.7	−529.5	2.81	−19.1	201.0	1419.9
13	4	10	28.1	33.1	−173.1	−139.6	-	−156.4	−49.9	−106.5	0.56	−3.8	40.4	284.4
**Pipe ø 300 mm–SMA wire ø 1 mm**														
14	4	0	29.6	31.9	−108.5	−105.4	−114.4	−109.5	−22.4	−87.1	0.39	−3.1	229.1	234.8
**Pipe ø 300 mm–SMA wire ø 2 mm**														
15	3	−2	23.0	23.1	−597.2	−760.5	−574.6	−644.1	−1.7	−642.4	2.88	−23.1	533.6	1738.3
16	4	−1	15.8	19.0	−482.3	−554.4	−458.0	−498.2	−32.4	−465.8	2.09	−17.9	429.3	1348.1
**Pipe ø 300 mm–SMA wire ø 3 mm**														
17	3	1	28.4	30.3	−153.8	−172.8	−177.8	−168.1	−18.1	−150.0	0.67	−5.4	57.7	407.3
18	4	9	25.8	5.1	−200.6	−266.1	−178.2	−215.0	206.6	−421.6	1.89	−15.2	162.0	1144.5

**Table 6:** Mean values of circumferential compression stress in concrete resulting from prestressing with SMA wire at the residual state σ_c,SMA_.

**Cylinder External Diameter**	**Mean Circumferential Compression Stress in Concrete Resulting from Prestressing with SMA Wire**		
	**ø 1 mm**	**ø 2 mm**	**ø 3 mm**
ø 200 mm	2 MPa	16 MPa	18 MPa
ø 250 mm	3 MPa	10 MPa	11 MPa
ø 300 mm	3 MPa	20 MPa	10 MPa

**Table 7:** Mean values of residual stress in SMA wire at the residual state σ_res_.

**Cylinder External Diameter**	**Mean Residual Stress in SMA Wire σ_res_**		
	**ø 1 mm**	**ø 2 mm**	**ø 3 mm**
ø 200 mm	194 MPa	378 MPa	192 MPa
ø 250 mm	276 MPa	235 MPa	120 MPa
ø 300 mm	299 MPa	478 MPa	110 MPa
Average	256.3 MPa	363.7 MPa	140.7 MPa

## Data Availability

The data presented in this study are available on request from the corresponding author.
